# Early newborn bathing practice and its determinants among postpartum women in Ethiopia: a systematic review and meta-analysis

**DOI:** 10.1186/s12884-024-06280-x

**Published:** 2024-01-27

**Authors:** Addis Eyeberu, Tamirat Getachew, Ayenachew Kebad, Adera Debella

**Affiliations:** 1 School of Nursing and Midwifery, College of Health and Medical Sciences, Haramaya University, Harar, Ethiopia; 2 School of Environmental Health, College of Health and Medical Sciences, Wollo University, Dessie, Ethiopia

**Keywords:** Early newborn bathing, Hypothermia, Postpartum women, Ethiopia

## Abstract

**Background:**

Early baby bathing has a major negative impact on the newborn's health. Even though early newborn bathing has numerous detrimental effects on neonatal health, evidence has provided little attention on the current level of practice. Furthermore, there is a dearth of data regarding the overall effects of early newborn bathing in Ethiopia, which would be helpful to program and policy designers. This meta-analysis aimed to ascertain the level of early bathing practice and its determinants among postpartum women in Ethiopia.

**Methods:**

All articles were searched from the Web of Sciences, CINAHL (EBOSCO), Science Direct, MEDLINE, PubMed, Google Scholar, and Google websites from inception to October 10, 2023. The meta-analysis was performed using Stata version 18. The summary estimates with 95% CI were estimated using the random effect model with the Der Simonian Liard method. Heterogeneity was explored using Galbraith plot, Cochrane Q statistics, I2 statistics, and test of theta. To deal with the observed heterogeneity, subgroup analysis, sensitivity analysis, and meta-regression were done.

**Results:**

This meta-analysis included a total of 2787 postpartum women. The pooled level of early newborn bathing practice among postpartum women in Ethiopia was 55% [95% CI: 38–71]. Based on subgroup analysis by region, the highest level of early newborn bathing practice was among studies conducted in the Afar region which was 73% (95% CI: 69–77). There is a significant association between maternal level of education and early newborn bathing practice among postpartum women in Ethiopia (AOR = 0.51, 95% CI: 0.24, 0.78).

**Conclusions:**

In this meta-analysis, the overall estimate illustrates that more than half of postpartum women practice early newborn bathing in Ethiopia. Maternal level of education was significantly associated with early newborn bathing practice. Thus, both the government and all the concerned stakeholders should take coordinated action to boost information dissemination and awareness creation among postpartum women thereby reducing the practice of early newborn bathing and alleviating consequences of early newborn bathing.

**Supplementary Information:**

The online version contains supplementary material available at 10.1186/s12884-024-06280-x.

## Introduction

Early newborn bathing is bathing the newborn before 24 h after delivery. The World Health Organization (WHO) recommends delaying bathing until 24 h after birth, and when not possible, to be delayed for at least 6 h [1]. Early newborn bathing is practiced across different regions of the world. The prevalence of early newborn bathing was 33.3% in Lebanon [2], 74.4% in Ghana [3], 74% in Malawi [4], 13% in Bangladesh [4], 32.5% in Jimma, Ethiopia [5], and 35.4% in Harar, Ethiopia [6]. Different literature showed that factors such as educational status, wealth, access to mass media, mode of delivery, and place of delivery were significantly associated with early newborn bathing practices [5–7].

Several types of research showed that delayed bathing has a paramount significance for newborns. Delaying the first bathing to more than 24 h after birth was effective in preserving the body temperature of the infant and improving moisture, then which may improve skin integrity and aid skin development [8]. When the body's temperature is preserved, it helps to avoid hypothermia-related morbidity [9]. It also allows the mother to have sufficient time for skin-to-skin contact which can improve breastfeeding, body temperatures, and blood glucose levels in neonates [2, 10]. In reverse, early bathing causes significant problems to the health of the newborn by destabilizing the vital signs and by causing to development of hypothermia, respiratory problems, and hypoglycemia [9, 11].

Neonatal mortality due to early bathing-induced hypothermia remains significant. A systematic review and meta-analysis study showed that neonatal mortality following early newborn bathing is significant compared to newborns with delayed bathing [12]. Another study also showed that mortality due to hypothermia in low-income countries was 15.4% [13]. However, other findings indicated that early newborn bathing increased the prevalence of hypothermia but not neonatal mortality [14].

To decrease the impact of early bathing, WHO recommends an appropriate time for the first bath in the package of postnatal care [15]. Even though immediate newborn care packages including counseling on when to bathe newborns are provided by health care providers immediately after delivery [16], early bathing is practiced by many postpartum women in Ethiopia. According to studies knowledge regarding early bathing is one of the contributing factors for such practice [6].

Although early newborn bathing is tedious and causes many negative impacts on newborn health, researchers have paid little attention to supporting the current practices in Ethiopia. In addition, there is a lack of evidence on the overall impact of early newborn bathing in Ethiopia which can be an input for policy and program design. Thus, this meta-analysis was aimed at determining the level of early bathing practice and risk factors among postpartum women in Ethiopia.

## Methods

### Protocol

This systematic review and meta-analysis study was done to determine the level of early bathing practice and its determinants among women in Ethiopia. The study is reported per Preferred Reporting Items for Systematic Reviews and Meta-Analyses (PRISMA) 2020 guideline (Supplementary file 1). The study is registered at PROSPERO with the registration number CRD42023493338.

### Eligibility criteria

The study utilized PICOS criteria. The population consisted of postpartum women who had given birth; the exposures are factors associated with the level of early newborn bathing practice; the outcome of the study was early newborn bathing practice; the studies' design were observational studies that looked at early infant bathing practices; and the context of the studies was Ethiopia. Additionally, the studies considered in this systematic review and meta-analysis study were written in English and from inception to October 10, 2023. This systematic review and meta-analysis excluded case series/reports, reviews, commentary, and editorials.

### Information sources

We performed intensive literature searches on different database platforms such as Web of Sciences, CINAHL (EBOSCO), Science Direct, MEDLINE, EMBASE, Google Scholar, and PubMed and websites until October 10, 2023. We also tried to access different universities' institutional repository websites. Unpublished articles were also searched directly on Google web search.

### Search strategy

The search was accomplished using a combination of Boolean logic operators (AND, OR, NOT), Medical Subject Headings (MeSH), and keywords. The search strategy for advanced PubMed includes "infant, newborn"[MeSH Terms] AND ("bathes"[All Fields] OR "bathings"[All Fields] OR "baths"[MeSH Terms] OR "baths"[All Fields] OR "bathe"[All Fields] OR "bathed"[All Fields] OR "bathing"[All Fields]) AND ("woman"[All Fields] OR "women"[MeSH Terms] OR "women"[All Fields] OR "woman"[All Fields] OR "women’s"[All Fields] OR "women's"[All Fields]) AND "Ethiopia"[MeSH Terms]. For other databases, keywords early newborn bathing AND Postpartum women AND Ethiopia were used.

### Study selection

All articles searched from the database and websites were consolidated, and exported to the reference management application (Endnote version X8). Then duplicate articles were manually removed. The titles and abstracts of the papers were then carefully evaluated. Two authors (AD, and AE) independently reviewed the full texts of the remaining publications to determine their eligibility based on predetermined inclusion and exclusion criteria (PICOS). The objectives, methodology, population, and significant findings (level of early newborn bathing practice, determinants, factors/predictors of early newborn practice) of the full-text studies written in English were then reviewed further. The two authors came to a logical agreement to handle any questions that developed during the extraction process, and the final agreement was finalized with the assistance of the authors (TG and AK). The PRISMA statement flow diagram is used to represent the entire research selection process.

### Data extraction

After identifying articles that met the inclusion, the two authors (AK and AE) extracted the data independently using a Microsoft Excel 2016 sheet. Different variables of interest were considered in the extraction and presented in table of summary. The accuracy of the data extraction was checked by comparing the results produced by the two authors. The information used for meta-analysis from the included articles was extracted, which included the sample size, frequency of the occurrence of early newborn practice, measure of associations with 95% CI, and effect size.

### Data item

The primary outcome of this meta-analysis was the level of early newborn bathing practice among postpartum women in Ethiopia. The early newborn bathing practice was defined as the practice of bathing a newborn 24 h after giving birth or before the WHO recommended time [17, 18] The secondary outcome of interest was identifying significant factors associated with Early newborn bathing practice among postpartum women in Ethiopia.

### Quality of study

The risk of bias was assessed using the Newcastle Ottawa scale (NOS) which is a validated tool for assessing the quality of non-randomized studies (cross-sectional studies) [19]. The risk of bias assessment tool includes the following domains: selection domain including representativeness of the sample, non-respondents, ascertainment of the exposure (risk factor); comparability domain (the subjects in different outcome groups are comparable, based on the study design or analysis and confounding factors are controlled); outcome domain such as assessment of the outcome and statistical tests. The included studies' methodological validity and the quality of their conclusions were scrutinized. Two authors (AD, and AE) assessed and scored the quality of the study using NOS. The mean score of the authors was utilized to make the final decision. Based on their performance against each tool indicator, the included studies were categorized as high, moderate, or low quality. The quality score of the 6 studies ranges from 6 to 7, with most studies (7 studies) scoring 7. All of the six studies were considered of adequate quality for inclusion in the analyses.

### Statistical analysis

Statistical analysis was conducted using STATA 18 software. The overall estimate of the level of early bathing and overall effect size of determinants with 95% CI were presented using forest plots. A meta-analysis of the level of early newborn bathing was done by random effect model using Der Simonian Liard method analysis to reduce the heterogeneity of the included studies. Since there was significant heterogeneity existed between included studies, subgroup analysis, sensitivity analysis, and meta-regression were performed to deal with such heterogeneity. Subgroup analyses were performed based on region and year of publication. Both bivariate and multivariate meta-regression were performed to determine and identify the source of heterogeneity. Sensitivity analysis was done using the leave-one-out meta-analysis method to identify the effect of a single study on the overall estimate and to identify the outliers. A meta-analysis of observational studies was undertaken based on the recommendations of the I2 statistic given by Higgins et al. (an I2 of 75/100% and above implying considerable heterogeneity). Additionally, a significant P value in the test of theta indicated heterogeneity between studies.

To look for potential publication bias, the researchers utilized Egger's Regression Test, trim fill analysis, and a visual evaluation of a funnel plot. Egger's test with a p-value less than 0.05 showed a small study effect on the estimate but an insignificant p-value indicates no small study effect. Trim fill analysis that showed no difference in observed and combination of observed and imputed effect indicated no publication bias. While we conducted a visual inspection of the funnel plot, the asymmetric presentation of the funnel plot indicated the presence of publication bias. Meta-analysis of factors associated with the level of early newborn bathing was analyzed using the random effect model by the Hedge method.

## Results

### Search finding and risk of bias assessment

**Fig. 1:**
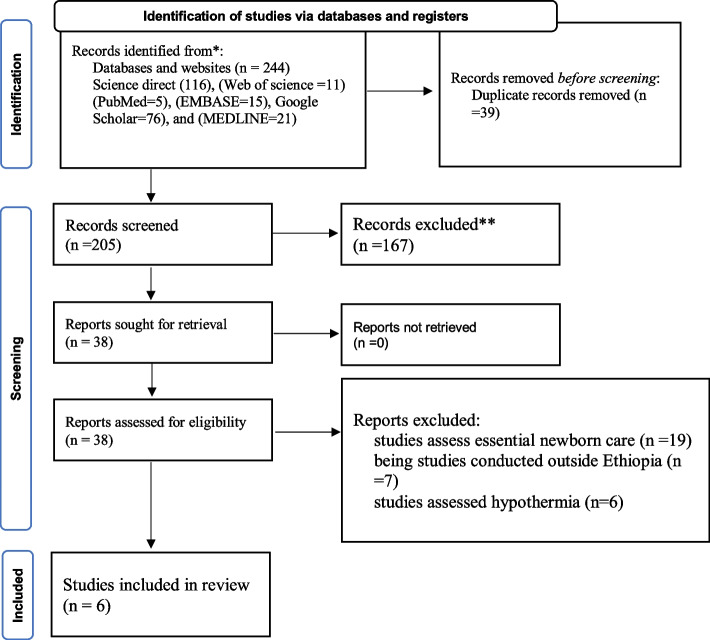
PRISMA 2020 flow diagram for systematic reviews and meta-analysis for early newborn bathing among postpartum women in Ethiopia, 2023

A total of 244 published and unpublished articles were found in legitimate databases and websites. Using ENDNOTE and visual inspection, 39 publications were removed from all identified studies due to duplication. The remaining 205 studies were then maintained and screened based on title and abstract. After being vetted based on titles and abstracts, 167 were removed. Thirty-eight articles were considered eligible for inclusion. Of those, 32 studies were removed due to reasons such as studies assessing essential newborn care practices, studies conducted outside Ethiopia, and studies assessed hypothermia. Finally, this systematic review and meta-analysis comprised six published observational studies that met the inclusion criteria (Fig. 1). Using the NOS criteria, all of the six studies yielded moderate and high-quality scores. Then all of the studies were considered eligible in this meta-analysis.

### Characteristics of included studies

This systematic review and meta-analysis comprised six observational studies (4 cross-sectional and two mixed studies) that assess early newborn bathing practice and associated factors. The sample size of the included studies ranges from a minimum of 382 in a study by Getachew, G. et al., [18] to a maximum of 610 in studies done by Girma, Tsinuel [20]. The majority of the included studies were published after 2022 [5, 17, 18, 21]. This illustrated that early newborn bathing is a recent concept and a current health problem for newborns. Of the included studies, half of the studies (3) were conducted in the Oromia region [5, 20, 21], one study from Harari [6], one study from Afar [18], and one study from the central Ethiopia region [17]. Three of the studies were conducted at the hospital while the other three studies were conducted in community settings. A total of 2787 postpartum women were included in this systematic review and meta-analysis. A summary of the main characteristics of the studies included in this systematic review and meta-analysis is presented in Table 1.

**Table 1:** Characteristics of included studies in this systematic review and meta-analysis in 2023

Author year	Journal	Setting	Region	Design	Sample size	Prevalence	Significant variables with 95 CI
Beyene D. A et al., 2023 [17]	SAGE Open Nursing	Community	Central Ethiopia	MS	582	43	(ANC) follow-up (AOR = 5.1 = .95% CI = (2.6–9.9)), having no recent complications during birth (AOR = 1.9 = 95% CI = (1.02–3.6), having information about the time of baby bathing (AOR = 6.02, 95% CI = (3.9, 9.3)), knowledge of hypothermia (AOR = 3.3 = 95.6% CI (1.9–5.8), and poor knowledge about neonatal danger signs
Fenta Kebede, B et al., 2022 [5]	Pediatric Health Med Ther	Hospital	Oromia	CS	388	32.5	Vaginal mode of delivery (AOR: 3.84 (95% CI: 1.96–7.52)), poor knowledge about danger signs (AOR: 6.78 (95% CI: 3.77–12.19), poor knowledge about hypothermia (AOR: 0.35 (95% CI: 0.20–0.58) and educational level of women (AOR: 0.33 (95% CI: 0.15–0.73)
Getachew, G. et al., 2023 [18]	Journal of Tropical Pediatrics	Hospital	Afar	CS	386	73.1	Mothers who attained college or higher education [adjusted odds ratio (AOR) ¼ 0.21; 95% CI 0.06–0.66], those who were from urban areas (AOR¼0.19; 95% CI 0.09–0.42) and those who gave birth using operational delivery (e.g. cesarean section and instrumental delivery) (AOR¼0.01; 95% CI 0.01–0.04)
Girma, Tsinuel, 2008 [20]	Ethiop J Health Sci	Community	Oromia	CS	610	58.4	
Wako WG et al., 2022 [21]	PLOS GLOBAL PUBLIC HEALTH	Community	Oromia	MS	388	84	
Welay, Fissaha T et al., 2020 [6]	The Open Public Health Journal	Hospital	Harar	CS	433	35.4	Uneducated (AOR = 3.12 95% CI: (2.12–5.3), no knowledge of hypothermia (AOR = 4.95 95% CI: (3.10–12.2), being primipara (AOR = 3.5 95% CI: (2.5–5.6) and no utilization of newborn bed net (AOR = 6.2 95% CI: (3.3–45)

*CS* Cross-sectional study, *MS* mixed study

### Meta-analysis of primary outcomes

**Fig. 2:**
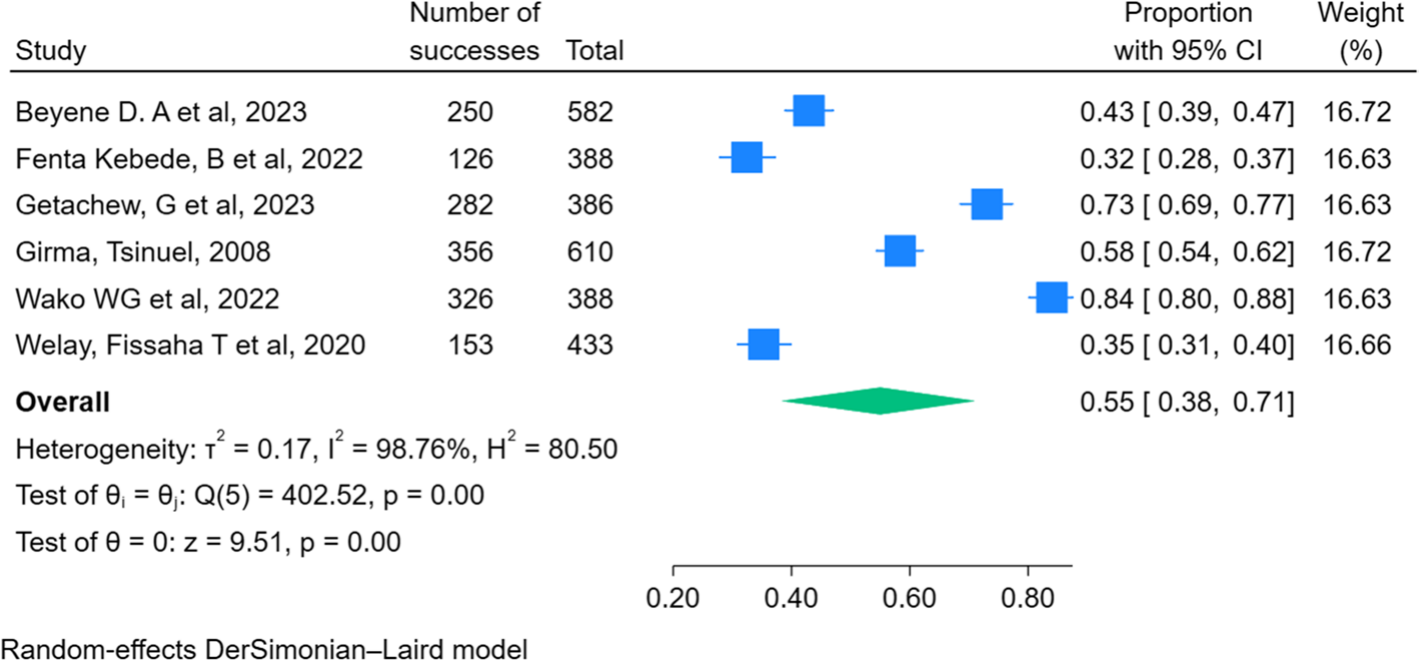
Pooled level of early newborn bathing practice among postpartum women in Ethiopia, 2023

All included studies were considered in a meta-analysis of the primary outcome of the study. The level of early newborn bathing practice ranges from 32% (95% CI: 28–37) to a maximum of 84% (95% CI: 80–88). The pooled level of early newborn bathing practice among postpartum women in Ethiopia was 55% [95% CI: 38–71]. There is significant heterogeneity among individual studies (i^2^ = 98.76%), the P value of the test of theta is also significant (P = less than 0.0001) and the confidence interval does not even overlap (Fig. 2).

### Subgroup analysis of the level of Early newborn bathing practice

**Fig. 3:**
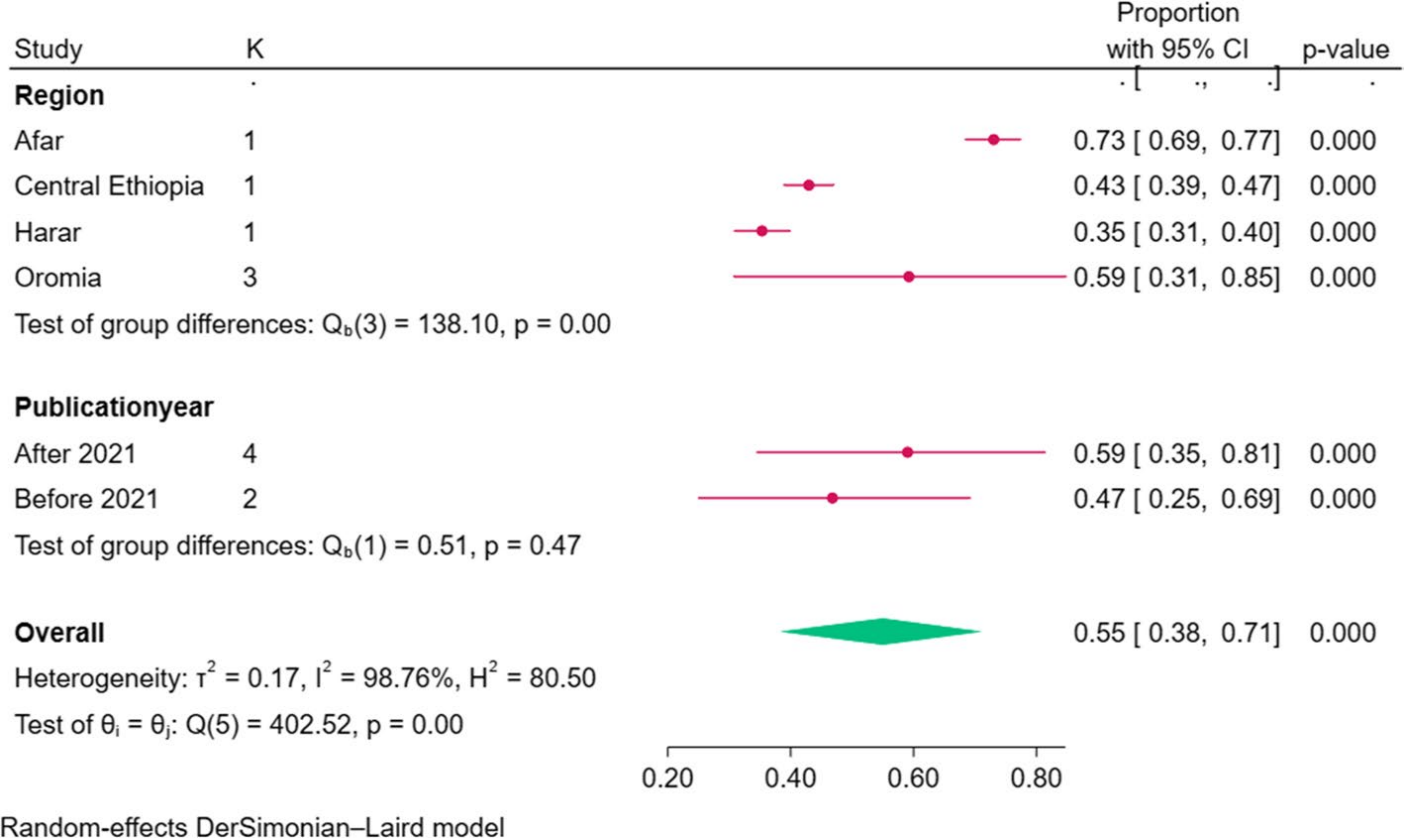
Subgroup analysis of the pooled level of early newborn bathing practice among postpartum women in Ethiopia based on region and publication year, 2023

Based on subgroup analysis by region, the highest level of early newborn bathing practice among postpartum women in Ethiopia was among studies conducted in the Afar region which was 73% (95% CI: 69–77). The heterogeneity among the subgroups was significant as the test of group difference showed that there is significant heterogeneity (P value less than 0.0001). Based on subgroup analysis by the publication year, the highest level of early newborn bathing practice among postpartum women in Ethiopia was observed among studies conducted during and after 2021 which was 59% (95% CI: 35–71) (Fig. 3).

## Publication bias

**Fig. 4:**
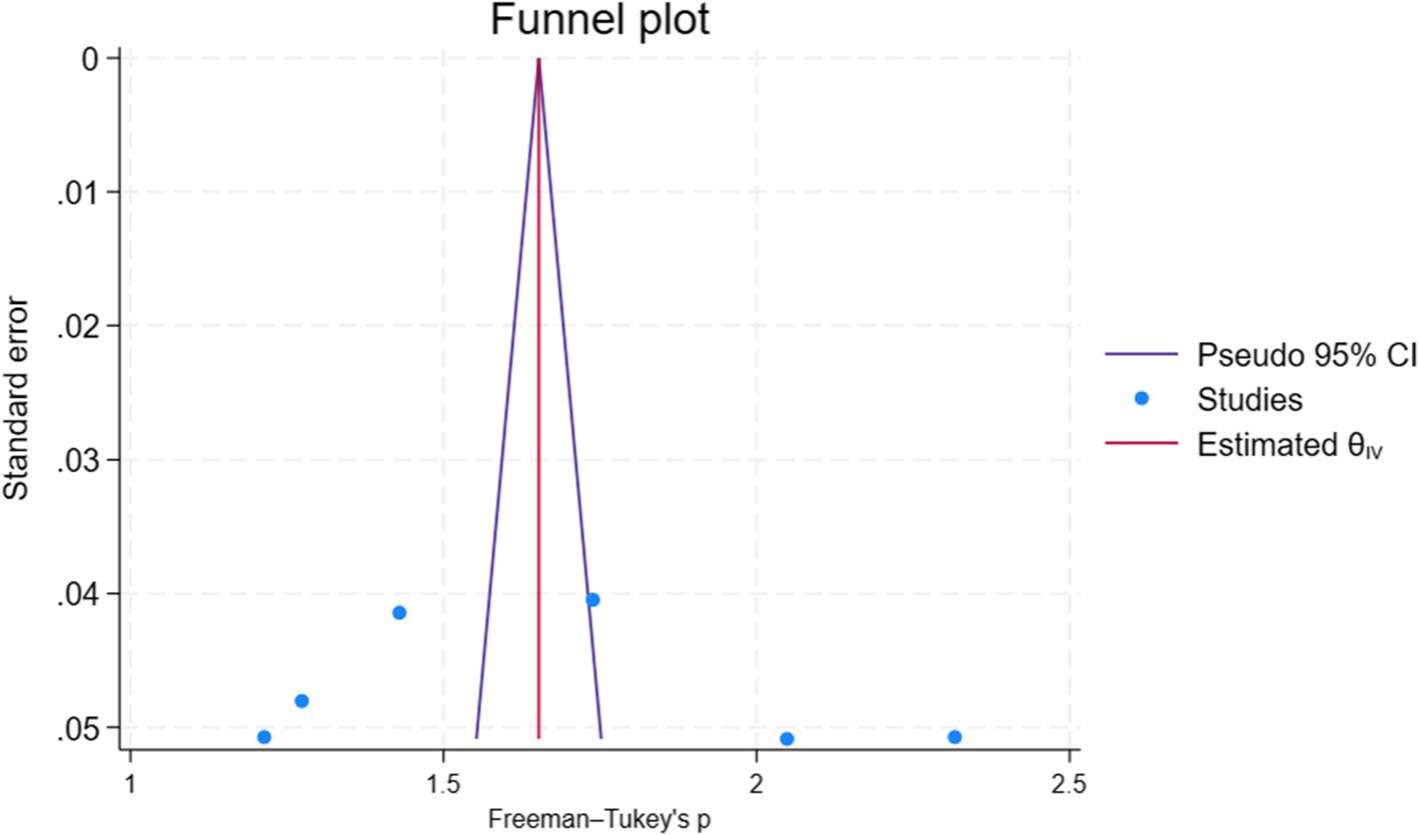
Funnel plot of the pooled level of early newborn bathing practice among postpartum women in Ethiopia, 2023

To observe publication bias, objective (statistical techniques) and subjective (a visual inspection) were carried out. A visual inspection of the funnel plot shows that there was asymmetry of the funnel plot and this may be due to significant heterogeneity existing among included studies (Fig. 4). An objective method of assessment of publication bias was also performed. Egger's test shows that there was no small study effect on the estimate (P = 0.87). Furthermore, Trim fill analysis also showed no difference in observed and combination of observed and imputed effect size estimate in the random effect model utilizing Der Simonian Liard.

## Multivariate meta-regression

The pooled level of early newborn bathing practice among postpartum women in Ethiopia shows that there was heterogeneity with Cochrane Q statistics, test of theta, and the I-square test statistics were significant. A meta-regression analysis was performed to identify the source of heterogeneity by considering variables sample size, region, year of publication, and study design. However, there is no variable found to be the source of the heterogeneity. Thus, other study-level covariates that were not considered in this analysis could be the source of heterogeneity (Table 2).

**Table 2:** Multi-variate meta-regression analysis of early newborn bathing practice among women in Ethiopia, 2023

Variables	Coefficients	Standard error	*P*	95% CI
Sample size	-0.0039292	0.0051149	0.442	-0.0139542, 0.0060958
Publication year	-0.0701271	0.0900382	0.436	-0.2465987, 0.1063444
Study design	-0.7467637	0.9245601	0.419	-2.558868, 1.065341
Region	0.0187053	0.4499933	0.967	-0.8632653, 0.9006759

## Meta-analysis of the factors associated with early newborn bathing

### The association between primary education (Vs no education) with early newborn bathing practice

**Fig. 5:**
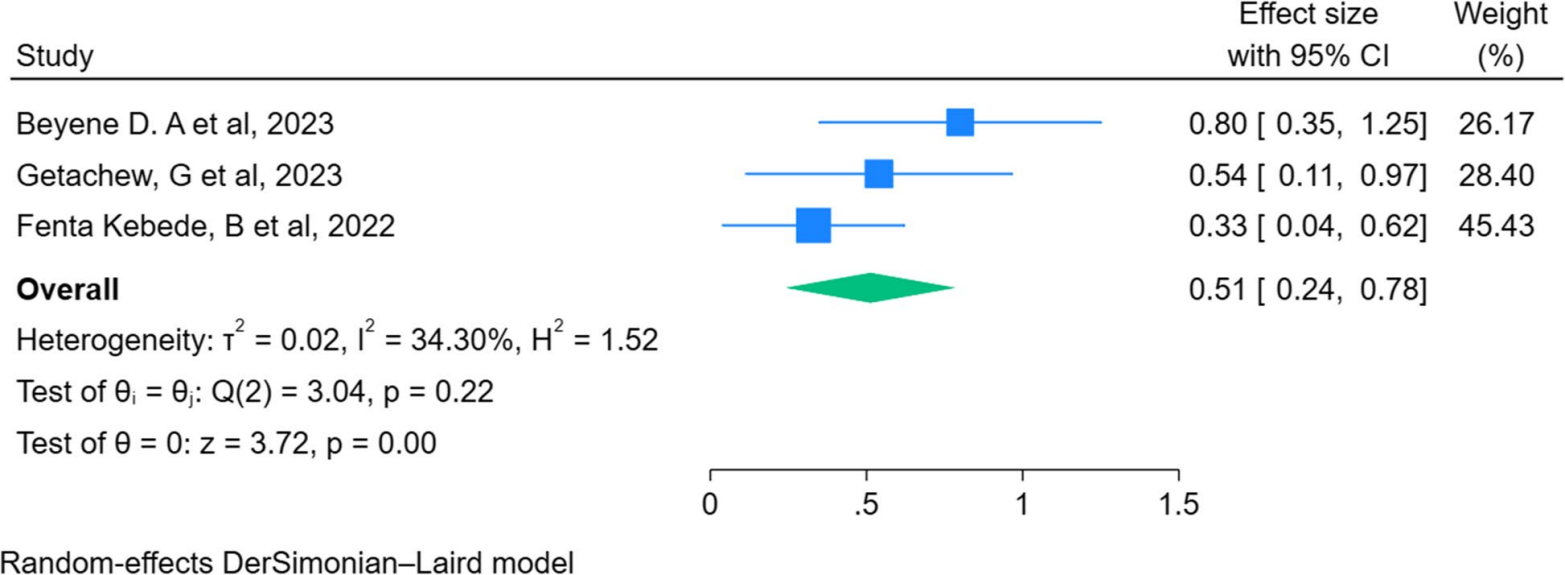
The association between primary education and early newborn bathing practice among women in Ethiopia, 2023

Of the six studies, three studies report the association between primary education and early bathing practices among women in Ethiopia. The overall pooled effect size showed that primary education is associated with early bathing practices among women in Ethiopia. Mothers who had primary education were 49% less likely to practice early newborn baths compared to mothers with no education (AOR = 0.51, 95% CI: 0.24, 0.78) (Fig. 5).

### The association between Antenatal care follow-up and knowledge of hypothermia with early newborn bathing practice

Of the total included studies, three studies examined the association between knowledge of hypothermia and early newborn bathing practice. The overall estimate showed that there was no statistically significant association between knowledge of hypothermia and early newborn bathing practice [OR = 2.33, 95%CI: -0.42–5.08] (Supplementary file 2)*.* Similarly, three studies examined the association between antenatal care visits and early newborn bathing practices. The overall estimate showed that there was no statistically significant association between having antenatal care visits and early newborn bathing practice [OR = 1.53, 95%CI: 0.34–2.71] (Supplementary file 3).

### Sensitivity analysis

**Fig. 6:**
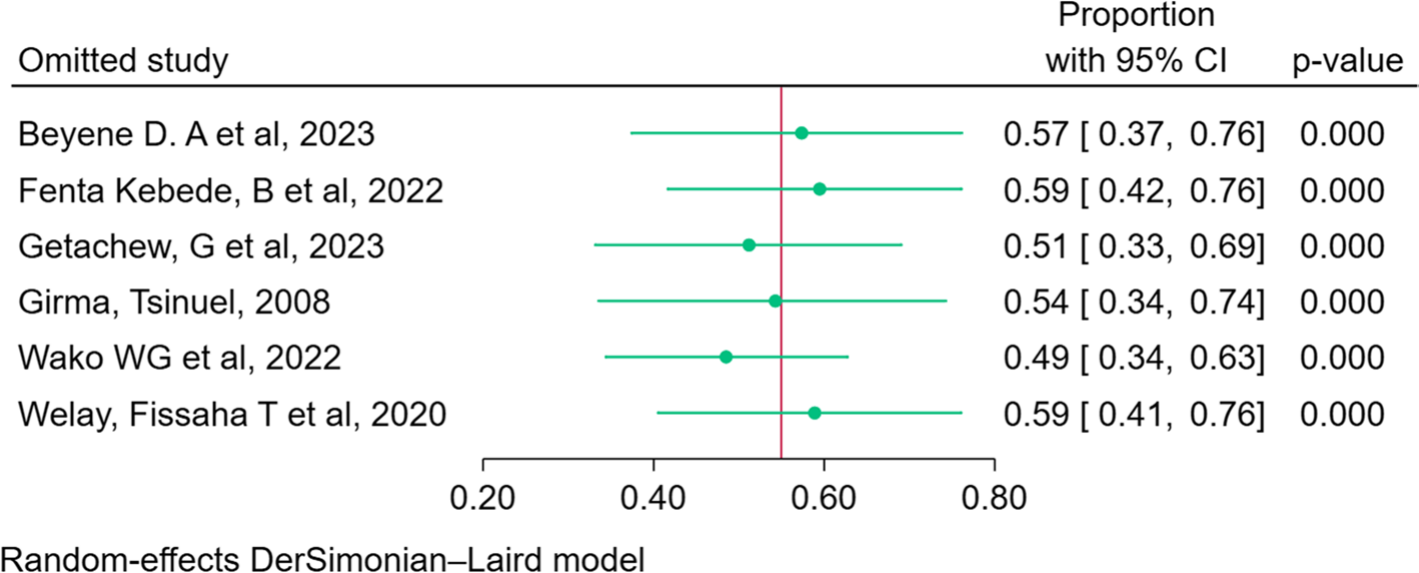
Leave one out a meta-analysis of the prevalence of early newborn bathing in Ethiopia, 2023

Leave one out meta-analysis using the Der Simonian Liard method was done to assess the effect of a single study on the overall estimate and to identify outliers. As shown in the figure below when individual studies were removed and the overall prevalence re-estimated, there was no such significant deviation from the prior determined prevalence (Fig. 6). However, the confidence interval of individual studies overlaps each other.

## Discussion

Even with the advancement of medical facilities to improve neonatal survival, hypothermia-related mortality and morbidity persisted. The two most typical effects of early newborn bathing practices are hypothermia and hypoglycemia. Despite efforts to improve newborn survival through the implementation of key newborn care packages., a lot of women are bathing their newborns too early, Thus, this systematic review and meta-analysis were aimed to determine the level of early newborn bathing practice among postpartum women in Ethiopia. In this review, the overall pooled level of early newborn bathing practice among postpartum women in Ethiopia was 55% [95% CI: 38–71]. Furthermore, maternal level of education was significantly associated with early newborn bathing practice among postpartum women in Ethiopia.

The results of this meta-analysis explicitly illustrate that more than half of postpartum women practice early newborn bathing in Ethiopia. This is consistent with a systematic review and meta-analysis study conducted in sub-Saharan Africa which showed that over 50% of women bathed their newborn early [22]. Similarly, a study done in Tanzania found that over two-thirds of women bathed their newborn early/ within 6 h [23]. This finding implied that even though bathing is important for newborns to keep and maintain skin integrity and texture, and protection against bacteria, the timing of bathing matters and it should be based on WHO recommendations. Early newborn bathing carries risks of hypothermia, respiratory compromise, and increased oxygen consumption. So, this study indicated that there is a gap in the implementation of immediate newborn care practice packages. Therefore, concerned bodies should strengthen a mechanism to increase immediate newborn care practices.

The finding of this study is much lower than a study done in Ghana 93% [24]. However, the finding is higher than studies conducted in Bangladesh 13% [4]. This implies that there are plenty of neonates who immensely suffer from the sequel of early newborn bathing practices. This implied that emphasis needs to be given to improve the awareness of the women regarding to timing of bathing. More efforts are also required from health care providers in promoting appropriate time of bathing.

This meta-analysis intensely reported that there is a significant difference in the level of early newborn bathing practice among regions of the country. Thus, the highest level of early newborn bathing practice among postpartum women in Ethiopia was among studies conducted in the Afar region which was 73% (95% CI: 69–77). Additionally, according to this systematic review and meta-analysis finding the lowest level of early newborn bathing practice among postpartum women in Ethiopia was among studies conducted in the Harari region which was 35% (95% CI, 31–40). This regional variation may be due to the difference in cultural beliefs regarding newborn care. A study done in the Oromia region showed that practice and beliefs about a delayed first bath are against WHO recommendations and it may be one reason for a high level of practice [21]. This study implied that coordinated and collaborative action is required from all concerned stakeholders to strengthen the existing standard of care practice, improving health education for women and training obstetrics care providers.

In this meta-analysis, maternal level of education was significantly associated with early newborn bathing practice. The overall pooled effect size showed that mothers who had primary education were 49% less likely to practice early newborn baths compared to mothers with no education (AOR = 0.51, 95% CI: 0.24, 0.78). This is crystal clear that those mothers with having primary level of education can understand the advantages and consequences of early newborn bathing. So, they prefer to delay bathing for at least 24 h. So, it is imperative to create awareness for women to delay bathing for at least 24 h after birth.

### Strengths and limitations of the study

The study's strength is that the publications were found in a variety of genuine databases, and most of them were recent publications. Another strength is that, to the best of the investigators' knowledge, it is the first SRM on the level of early newborn bathing practice and associated factors among postpartum women in Ethiopia. However, the majority of the included studies came from a few regions. During the article search, only the English language was taken into account, which may influence the overall estimate.

## Conclusion

In this meta-analysis, the overall pooled level of early newborn bathing practice among postpartum women in Ethiopia was 55% [95% CI: 38–71]. This overall estimate illustrates that more than half of postpartum women practice early newborn bathing in Ethiopia. Maternal level of education was significantly associated with practicing early newborn bathing. Thus, both the government and non-governmental stakeholders should take coordinated action to boost information dissemination and awareness creation among postpartum women thereby reducing the practice of early newborn bathing and alleviating consequences of early newborn bathing.

### Supplementary Information

**Additional file 1**: **Supplementary file 1.** PRISMA 2020 flow. **Supplementary file 2.** The association between knowledge and early newborn bathing practice among women in Ethiopia, 2023. **Supplementary file 3.** The association between ANC and early newborn bathing practice among women in Ethiopia, 2023.

## Data Availability

In this study, all pertinent information is given. However, the corresponding author will provide more information upon reasonable request.
